# Complete genome sequence of bacteriophages Merry and Sunny infecting *Microbacterium chocolatum* strain *GAI20246-6* isolated from an outdoor commercial algal pond

**DOI:** 10.1128/mra.00767-24

**Published:** 2025-10-20

**Authors:** Alice V. Levesque, Ariel J. Rabines, Entesar Alrubaiaan, Aaron Oliver, Eric E. Allen, Dave Hazlebeck, Aga Pinowska, Jesse C. Traller, Lisa Zeigler Allen

**Affiliations:** 1 Scripps Institution of Oceanography, University of California San Diego 8784, La Jolla, California, USA; 2 Global Algae Innovations, Lihue, Hawaii, USA; Portland State University, Portland, Oregon, USA

**Keywords:** microbacteriophage, viral diversity, algae aquaculture

## Abstract

We report the isolation of two virulent phages from an outdoor algal pond infecting the bacteria *Microbacterium chocolatum* strain GAI20246-6. Their genomes are both 53.6kb with a GC content of 67.8%. Some genomic features are described as well as morphological characteristics based on transmission electron microscopy (TEM) micrographs.

## ANNOUNCEMENT

Outdoor algal ponds are a sustainable alternative to produce various biomolecules (1). At Global Algae Innovations (21.996N 159.375W, Lihue, HI, USA), *Nannochloris sp.* (Chlorophyta) is used and develops an associated microbiome. *Microbacterium chocolatum* strain *GAI20246-6* was isolated from these ponds (2) and demonstrated algicidal properties in laboratory conditions when co-cultivated with *Nannochloris sp*. We sought to use phage therapy as a strategy to regain algal productivity, thus demonstrating the use of microbacteriophage within commercial applications. Using *M. chocolatum* strain *GAI20246-6* as host for all assays, two phages were isolated.

Using the double-layer method, 50× concentrated (Vivaflow 50R, Sartorius) surface pond water (July 2018–January 2019) was spotted onto a host lawn (30°C Tryptic Soy Agar (TSA) 48 h). Clear viral plaques, indicative of a virulent phage, were isolated from two different samples, and a single clonal population was obtained following three rounds of purification (3). Liquid phage propagations were conducted in Tryptic Soy Broth (TSB) and incubated overnight at 30°C (200 rpm). For TEM, phage particles were stained (2% uranyl acetate) on a grid (Formvar/Carbon 200 mesh TH Copper, Ted Pella) and observed on a JEOL 1400 instrument. DNA was extracted from a phage lysate using the NucleoMag Virus kit (Machery-Nagel) and treated with RNase A (Thermo Scientific, 10 mg/mL). DNA was fragmented for size selection (Covaris S220), and libraries were constructed using Accel-NGS 2S PCR-free DNA library kit (Swift Biosciences), including eight PCR cycles (Kapa HiFi PCR, Kapa Biosystems) due to low DNA input (<10 ng). Sequencing was performed on Illumina MiSeq v2 500 cycles (2 × 150 bp). Reads were trimmed (Trimmomatic v1.2.15 [4]) for quality (minimum score 33) and to remove Illumina adapters (minimum internal and terminal hit length of 10). Viral genomes were assembled using CLC Genomics Workbench (clc_assembler) followed by VirSorter2 (5) and CheckV 1.0.1 (6). Annotation was conducted with Pharokka 1.7.1 (7), where CDS are predicted using Pyrodigal-gv 0.3.1 (8, 9) and PHANOTATE 1.5.1 (10), and tRNAs using tRNAscan-SE 2.0 (11) and Aragorn 1.2.41 (12). Each CDS function was assigned using the PHROG (13), VFDB (14) and CARD (15) databases within MMseq2 (16) and PyHMMER (17). Final annotation was manually curated, and five pairs of primers were designed to confirm genomic termini regions with PCR (2× Taq RED Master Mix, Apex). A repeated motif within both genomes was detected using Geneious Prime 2024.0.4 and aligned with MUSCLE 5.1 (18). Default parameters were used except where otherwise noted.

**Fig 1:**
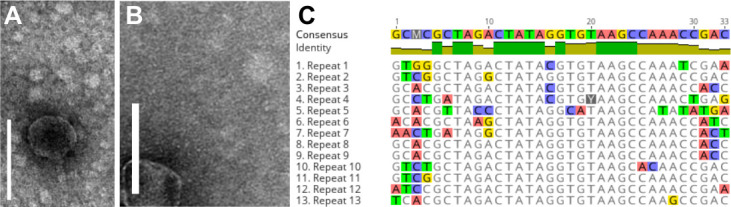
TEM micrographs of phage Merry (**A**) and Sunny (**B**) (5′-revealed a siphovirus morphology with icosahedral capsid and long flexible tail. Scale bar is 100 nm. Motif gcMcGcTAgaCTATAgGtgtAAGCcaaaccgac-3′) found 13 times throughout the phage genomes (capital letter conserved in all repeats, lower-case letter = conserved in at least 6/13 repeats, M=A/C are equal) (**C**). The “Y” in repeat 4 indicates a substitution of T>C at position 20 of the consensus sequence, distinguishing Sunny (T) and Merry (**C**).

TEM revealed siphovirus morphology (Fig. 1A and B). BLASTn (19) showed similarity to the genera *Quhwahvirus* and *Metamorphoovirus* (Table 1). Genomic features are summarized in Table 1. The genomes differ (nucleotide identity 89%) in a 1 kb region comprising a putative head-tail adaptor gene. Both phages are complete with a circularly permuted genome confirmed through PCR. Phages were assigned to Microbacteriophage Cluster EC based on genome similarity (Table 1) (https://phagesdb.org, accessed on May 2024 [20, 21]) and shared features, such as possessing phage-encoded glycosyltransferase and UDP-glucose dehydrogenase genes, and a conserved 33 bp motif that is repeated 13 times throughout their genome in intergenic regions (Fig. 1C).

**TABLE 1:** Bacteriophage names, morphology, and genome characteristics

Phage ID	Morphology^*a*^		Reads^*b*^	Coverage (fold)	Genome (bp)	GC content (%)	No. of tRNAs	CDS	Function annotated	Coding strand^*d*^	BLASTn results	
	Capsid size	Tail length	Post-QC								Top 3 best match	Nucleotide identity
	(nm)	(nm)									*Microbacterium* phage^*c*^	
Merry			1,628,150	5,132	53,640	67.8	0	93	24	+	Jayden NC_073375	57.09%
	63.63	148.36									Fireman MK524510.1	55.97%
	(±2.94)	(±11.11)									Metamorphoo MH271304	54.97%
Sunny			1,445,364	3,691	53,642	67.8	0	93	24	+	Jayden NC_073375	56.92%
	72.43	145.89									Fireman MK524510.1	55.89%
	(±3.81)	(±7.24)									Quhwah MH271321	55.28%

^*a*^ Capsid size and tail length are averages (*n* = 18) with standard deviation of population means.

^*b*^ Raw reads: Merry 1,736,606, Sunny 1,478,076.

^*c*^ Identified using BLASTn 2.2.26 (15) against the Actinobacteriophage database (https://www.phagesdb.org, accessed on May 2024). CDS = coding sequence, tRNA = transfer RNA, BLASTn = Nucleotide BLAST (Basic Alignments Search Tool). Merry was isolated from GAI pond water collected on 1 August 2018, and Sunny on 20 January 2019.

^*d*^ "+” indicates coding strand.

## Data Availability

Phage raw reads have been deposited in the NCBI Sequence Read Archive (accession numbers SRR28924545 and SRR28924546, under BioProject PRJNA1107628), and the annotated genomes have been deposited in GenBank (accession numbers PP763431 and PP763432). *Microbacterium chocolatum* strain *GAI20246-6* is available in GenBank (BioProject PRJNA1129581 and accession number JBEVYX000000000). The strain used in this study is available upon request.
