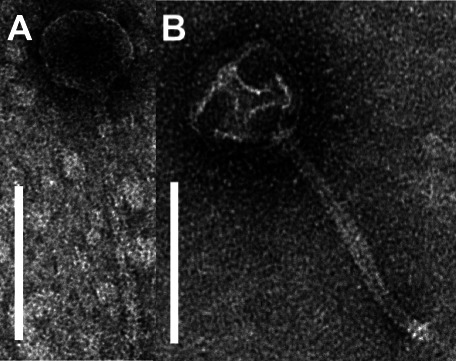# Erratum for Levesque et al., “Complete genome sequence of bacteriophages Merry and Sunny infecting *Microbacterium chocolatum* strain GAI20246-6 isolated from an outdoor commercial algal pond”

**DOI:** 10.1128/mra.01319-25

**Published:** 2026-02-26

**Authors:** Alice V. Levesque, Ariel J. Rabines, Entesar Alrubaiaan, Aaron Oliver, Eric E. Allen, Dave Hazlebeck, Aga Pinowska, Jesse C. Traller, Lisa Zeigler Allen

## ERRATUM

Volume 14, no. 11, e00767-24, 2025. https://doi.org/10.1128/mra.00767-24. "*GAI20246-6*" should appear as "GAI20246-6" in the title and throughout the article.

Figure 1: Panels A and B should appear as shown in this erratum. Formatting issues caused the TEM micrographs of phages Merry and Sunny to get displaced within the table, which resulted in the problem observed in the published article. We apologize for this formatting error.

In the Fig. 1 caption, “…Sunny (**B**) (5′-revealed a siphovirus morphology with icosahedral capsid and long flexible tail. Scale bar is 100 nm. Motif gcMcGcTAgaCTATAgGtgtAAGCcaaaccgac-3′)” should read “…Sunny (**B**) revealed a siphovirus morphology with icosahedral capsid and long flexible tail. Scale bar is 100 nm. Motif (5′-gcMcGcTAgaCTATAgGtgtAAGCcaaaccgac-3′)…”

**Fig 1:**